# Detoxification of aflatoxin B1 by a *Bacillus subtilis* spore coat protein through formation of the main metabolites AFQ1 and epi-AFQ1

**DOI:** 10.3389/fmicb.2024.1406707

**Published:** 2024-10-04

**Authors:** Raditya Subagia, Wolfgang Schweiger, Elisavet Kunz-Vekiru, Dominik Wolfsberger, Gerd Schatzmayr, Doris Ribitsch, Georg M. Guebitz

**Affiliations:** ^1^Department of Agrobiotechnology (IFA-Tulln), Institute of Environmental Biotechnology, University of Natural Resources and Life Sciences, Vienna, Austria; ^2^dsm-firmenich, Animal Nutrition and Health R&D Center Tulln, Tulln, Austria

**Keywords:** mycotoxins, laccase, enzymatic degradation, genotoxicity, copper incorporation

## Abstract

A variety of important agricultural crops host fungi from the *Aspergillus* genus can produce cancerogenic secondary metabolites such as aflatoxins. Consequently, novel strategies for detoxification and their removal from food and feed chains are required. Here, detoxification of Aflatoxin B1 (AFB1) by the *Bacillus subtilis* multi-copper oxidase CotA (BsCotA) was investigated. This laccase was recombinantly produced in *E. coli* while codon optimization led to duplication of the amount of active protein obtained. CuCl_2_ was added to the cultivation medium leading to a 25-fold increase of *V*_max_ corresponding to improved incorporation of Cu^2+^ into the enzyme protein which is essential for the catalytic reaction. To avoid potential cytotoxicity of Cu^2+^, cultivation was performed at microaerobic conditions indeed leading to 100x more functional protein when compared to standard aerobic conditions. This was indicated by an increase of *V*_max_ from 0.30 ± 0.02 to 33.56 ± 2.02 U/mg. Degradation kinetics of AFB1 using HPLC with fluorescence detection (HPLC-FLD) analysis indicated a theoretical substrate saturation above solubility in water. At a relatively high concentration of 500 μg/L, AFB1 was decomposed at 10.75 μg/Lh (0.17 nmol^*^min^−1*^mg^−1^) at a dosage of 0.2 μM BsCotA. AFQ1 and epi-AFQ1 were identified as the initial oxidation products according to mass spectrometry (i.e., HPLC-MS, HPLC-QTOF). None of these molecules were substrates for laccase but both decomposed in buffer. However, decomposition does not seem to be due to hydration of the vinyl ether in the terminal furan ring. Genotoxicity of the formed AFB1 was assessed in several dilutions based on the de-repression of the bacterial SOS response to DNA damage indicating about 80-times reduction in toxicity when compared to AFQ1. The results of this study indicate that BsCotA has high potential for the biological detoxification of aflatoxin B1.

## 1 Introduction

Aflatoxins are cancerogenic secondary metabolites produced by several *Aspergillus* species, predominantly *A. flavus* and *A. parasiticus* (Fouché et al., 2020). A number of agricultural relevant crop species such as corn and peanuts are common hosts to these fungi, leading to frequent contaminations throughout the globe, but predominantly in warm and humid climates. Aflatoxins and their derivatives share a planar difuranocoumarin core structure with partially strong affinities to DNA (Stone et al., 1988; Neto and Lapis, 2009; Mariën et al., 1987). These vary considerably depending on how well the distinct molecular structures can intercalate with DNA (Iyer et al., 1994; Stone et al., 1988). Aflatoxins are activated into a short-lived *exo*-8,9-epoxide via oxidation of the vinyl ether in the terminal furan ring by liver P450-dependent monooxygenases (Shimada and Guengerich, 1989). The highly reactive metabolite readily forms covalent bonds to the guanine N7 of DNA, which further reacts to a formamidopyrimidine adduct (Klvana and Bren, 2019; Croy et al., 1978; Essigmann et al., 1977). Aflatoxin B1 induces misincorporations during DNA replication, in particular the guanine to thymine transversion in the human p53 tumor suppressor gene, which is linked to the initiation of hepatocarcinoma formation (Aguilar et al., 1993). Aflatoxin B1 (AFB1) is the predominant metabolite and the most toxic representative of aflatoxins, and while the contamination levels are generally much lower than for other frequent toxic fungal contaminants on crops such as deoxynivalenol or fumonisin B1, chronic exposure is associated with higher risk of development of liver cancer. Consequently, very low maximum levels in food and feed stuffs have been issued by all major authorities (EUR-Lex - 02006R1881-20140701 - EN - EUR-Lex, 2023).

Strategies to remove AFB1 from the food and feed chain have been studied extensively. AFB1 can be decomposed by physical and chemical methods, such as treatment with hydrogen peroxide or ammonium (Rushing and Selim, 2019). However, neither these approaches are cost-effective, nor they are well-applicable in industry. Silicate-based binders can absorb AFB1 in feed, but the efficacy highly depends on the chemical composition and administration dosage of the respective product (Vekiru et al., 2015). Alternatively, a number of reports exist on biological degradation of AFB1 by microbes reported from a plethora of different genera (Mishra and Das, 2003; Vanhoutte et al., 2016; Taheur et al., 2020; Mwakinyali et al., 2019; Li et al., 2018; Arimboor, 2024; Gu et al., 2024; Wu et al., 2024; Tang et al., 2024). However, most of these reports lack the experimental details to unambiguously document biological/enzymatic degradation in contrast to frequently observed absorption effects. Enzymes as biocatalysts allow detoxification of AFB1 in food and feed products at mild conditions (temperature, pH) and are highly specific which avoids potential damage of valuable ingredients that could happen with other treatments (e.g., high temperature). Only recently, the degradation of aflatoxin B1 by the lipase and protease of *Humicola lanuginosa* was reported (Al-Rajhi et al., 2024). Enzymatic degradation is well-documented for members of the phyla *Actinomycetales* (Ciegler et al., 1966; Teniola et al., 2005) in which F_420_H_2_-dependent reductases catalyze the reduction of a double bond in the AFB1 lactone core, which enables further decomposition of the otherwise rigid molecular structure (Bashiri, 2022; Taylor et al., 2010). Other reports on enzymatic degradation refer to manganese peroxidases (Wang et al., 2019a, 2011; Loi et al., 2020) and laccases predominantly from filamentous fungi (Alberts et al., 2009; Guo et al., 2020). Manganese peroxidases transfer electrons from hydrogen peroxide to reduce Mn^2+^. AFB1 degradation by peroxidases thus again requires addition of hydrogen peroxide and hence may be more a function of the initial peroxide concentration in the assay, than related to an enzymatic process.

Laccases (EC 1.10.3.2) are multicopper oxidases which can act on a broad range of substrates (Giardina et al., 2010; Pardo and Camarero, 2015). The ability to oxidize a substrate is limited by steric and electrostatic hindrance but appears to primarily depend on the enzymes specific redox potential presented by a type 1 (T1) copper, which acts as a primary acceptor of electrons from the ligand (Tadesse et al., 2008; Xu et al., 1998). A general classification organizes laccases into high redox potential enzymes mostly from fungi such as *Trametes versicolor* (~780 mV) and medium/low redox potential laccases originating from plants and prokaryotes. High laccase activities in several fungal culture supernatants were associated with the higher ability to degrade AFB1 and a commercial batch of *T. versicolor* laccase did degrade AFB1 into a non-toxic product (Alberts et al., 2009). However, laccases can not only convert AFB1, but also the metabolites AFB2, AFG1 and AFG2, as molecular docking studies have indicated using the laccase from *Saccharomyces cerevisiae* (Liu et al., 2020). Additionally, Loi et al. (2016) assessed the degradation of AFM1 by Lac2 from fungi *Pleurotus pulmonarius*. The activity in AFB1 is not restricted to high redox potential laccases. High redox compounds were oxidized at higher rates by the low redox potential CotA laccase of *Bacillus subtilis* (525 mV) (Martins et al., 2015).

In 2019 (Kunz-Vekiru et al., 2019), we have shown that the laccase CotA from *Bacillus subtilis* is able to degrade AFB1 without the need for mediators. Mediators are small organic compounds that can act as electron carriers between the enzyme and the substrate. Oxidation of the mediator by laccase leads to the formation of highly active cation radicals that oxidize compounds that laccase alone cannot oxidize (Munk et al., 2018). Albeit using mediators, in the same year, likewise Wang et al. (2019b) reported a decrease of the AFB1 concentration upon incubation with this laccase. However, this activity was based on the decrease of AFB1 determined by HPLC while potential degradation products were not identified. Afterwards, other working groups have reported the activity of a different CotA enzymes from another *Bacillus* species, namely *B. licheniformis* on AFB1 (Wang et al., 2019b; Sun et al., 2023; Liu et al., 2023; Wang et al., 2024; Guo et al., 2020). However, this enzyme shows very low identity (65%) and homology (78%) to CotA from *B. subtilis*. Hence, it would be interesting to elucidate to which degradation products these different laccases lead and how this laccase could potentially be produced in higher yields. The results could provide important insights into how strongly the activity on AFB1 is conserved in *Bacillus*. Therefore, in this study, we have studied in detail the catalytic detoxification of AFB1 by the recombinantly produced and purified *B. subtilis* CotA (BsCotA) laccase without the use of mediators. For the first time, aspects are highlighted that have previously received only minor or no attention, such as gene design and expression optimization for the best possible incorporation of copper into the laccase or the instability of the degradation product AFQ1. We have identified aflatoxin Q1 (AFQ1) and its epimere (epi-AFQ1) as the main reaction products by LC-MS and LC-HRMS and determined the residual mutagenicity compared to AFB1.

## 2 Materials and methods

### 2.1 Materials

LB media premix, containing 10 g/L tryptone, 5 g/L yeast extract, 5 g/L sodium chloride (NaCl), for culturing *E. coli* BL21-Gold (DE3) was purchased from Roth, Germany. The following chemicals (all purchased from Sigma-Aldrich, Germany) were dissolved in Milli-Q and sterile filtered: 50 mg/mL kanamycin, 1 M CuCl_2_ and 1M IPTG. AFB1 powder was purchased from Fermentek (Jerusalem, Israel) and dissolved in acetonitrile to a stock solution of 900 mg/mL. AFQ1 and epi-AFQ1 were purchased from Toronto Research Chemicals (Toronto, Canada) as solid substances and dissolved to 250 and 100 μg/mL stock solutions, respectively, in 100% acetonitrile.

LC gradient grade acetonitrile as well as formic acid (p.a.) were purchased from Merck (Darmstadt, Germany). A Purelab Ultra system (ELGA LabWater, Celle, Germany) was used for further purification of reverse osmosis water. 2,2′-azino-bis(3-ethylbenzothiazoline-6-sulfonic acid) (ABTS) was obtained as solid substance from Roche, Germany and dissolved to 10 mM in water.

### 2.2 Cloning, expression, purification and stabilization of CotA

A codon optimized version of the gene encoding the *Bacillus subtilis* CotA (Uniprot P07788) was synthesized in fusion to a C-terminal Strep-tag II (GenScript, Piscataway, NJ, USA) and cloned downstream of the T7 promoter into the *Escherichia coli* expression vector pET26b(+) over the NdeI and HindIII restriction sites. Chemically competent *E. coli* BL21-Gold (DE3) cells (Invitrogen, Carlsbad, CA, USA) were transformed with the plasmid and the laccase expression induced by addition of IPTG. Therefore, freshly transformed cells were cultured overnight at 37°C and 150 rpm (HT Multitron Pro, Infors, Bottmingen, Schweiz) in LB media containing 40 μg/mL kanamycin. The overnight culture was used to inoculate 300 mL LB media supplemented with 40 μg/mL kanamycin to a starting OD_600_ of 0.1 in a 1 L shake flask. Cells were grown aerobically at 30°C and 120 rpm until an OD_600_ of 0.6 was reached, after which the incubation temperature was reduced to 25°C followed by addition of 0.25 mM CuCl_2_ and 0.05 mM IPTG to the culture media. Aerobic incubation was continued further for 4 h, and the change to microaerobic conditions was achieved by switching off the shaking function of the incubator. Cells were harvested after 20 h of microaerobic expression by centrifugation at 3,700 rpm for 20 min at 4°C (Eppendorf centrifuge 5920 R, Germany). 1 g cell pellet was resuspended in 5 mL buffer W (100 mM Tris/HCl pH 8.0 and 150 mM NaCl) and disrupted by sonication (Digital sonifier, Branson, CT, USA) by applying 10 rounds × 10 s pulses of 60% amplitude with 2 min rest between rounds. The lysate was centrifuged again, and sterile filtrated.

BsCotA_Strep was purified by affinity chromatography (Äkta pure, GE Healthcare, Chalfont St. Giles, UK) from the cleared lysate according to manufacturer's protocol (IBA GmbH, Göttingen, Germany). Briefly, 20 mL of the cleared lysate were loaded onto a 1 mL Strep-Tactin XT cartridge. The column was washed with buffer W (1 mL/min) until the baseline is reached and the enzyme was eluted during a 3-column volume gradient from 0 to 100% of buffer BXT (100 mM Tris/HCl pH 8.0, 150 mM NaCl and 50 mM biotin). Buffer was exchanged to 100 mM Tris/HCl pH 7 using PD-10 desalting columns (GE Healthcare, Chalfont St. Giles, UK) and the protein was stored at −20°C until further use. To improve enzyme stability, bovine serum albumin (BSA) was added making an end concentration of 2 mg/mL in the storage buffer. BsCotA_Strep expression levels were analyzed by SDS PAGE, and protein concentrations calculated from the absorbance values obtained at 280 nm via molecular extinction coefficient of 93,740 M^−1^ cm^−1^ and molecular weight of 59.831 kDa (NanoDrop Spectrophotometer NP80 Touch, Implen, München, Germany).

Untagged BsCotA was expressed in the same way as BsCotA_Strep but purified by cation exchange chromatography. By using a washing buffer (20 mM Tris HCl pH 7.6) with a pH lower than the theoretical pI of CotA (pI 7.7), the laccase became positively charged and attached to the cation exchanger column (GE HiTrap SP FF 1 mL). Subsequently, elution was carried out with 20 mM Tris HCl pH 7.6 containing 1 M NaCl. After the pellet was resuspended in washing buffer and extracted with a sonicator (10 cycles of 10 s at 60% amplitude with 2 min rest between runs), the cell lysate was centrifuged for 25 min at 20,817 × g. The resulting cell lysate was then filtered and loaded onto the column at a flow rate of 1 mL/min. Following sample loading, the column was further washed with washing buffer to remove nonspecifically bound proteins, indicated by a decrease in UV absorbance at 280 nm. Elution of additional impurities was achieved by applying elution buffer at 10% for 10 column volumes (CV), while elution of the target protein occurred by applying elution buffer as a gradient toward 50% over 10 CV. Finally, 100% elution buffer was applied for 10 CV to remove any remaining attached proteins.

### 2.3 Determination of copper incorporation rates

The emitted signal of the complex formed between reduced copper released by the BsCotA protein with Bicinchoninic acid (BCA) was photometrically measured at two different wavelengths. The Cu(I)BCA complex is generally read at 562 nm at which the reading is exceptional tolerant to non-ionic detergents, competing cations, a variety of buffer salts and organic compounds, while at 359 nm the sensitivity of BCA is about 2.5 times greater than commonly used reagents for colorimetric determinations of copper (Brenner and Harris, 1995). A series of standard dilutions ranging from 200 to 300 μg/dL, and BsCotA concentration of 1 mg/mL was processed according to the copper assay kit protocol from Sigma-Aldrich, Germany. Calculation using higher standard in comparison to copper concentration of the sample was suggested by the manufacturer.

### 2.4 ABTS standard activity assay

BsCotA and BsCotA_Strep activity was quantified on the model substrate ABTS using a setup consisting of 20 μL enzyme (Vol_Enyzme_), 150 μL buffer (not oxygen saturated), and 50 μL ABTS (10 mM). The components were consecutively added, and the reaction started by the addition of ABTS. The increase of absorbance of oxidized ABTS per min (ΔAbsorbance) at 420 nm in 96 well plate was photometrically measured at 405 nm at room temperature (Plate Reader Tecan Infinite M200 Pro, Switzerland). In order to achieve a linear increase of oxidized ABTS, CotA stock solution of 5 mg/mL (C_Enzyme_) was diluted 400 times (df) before added to the setup, representing BsCotA concentration of 0.0189 μM in the end volume of 220 μL (Vol_End_). Volumetric and specific activity were calculated with the following formula, via ε [molar extinction coefficient of ABTS in 100 mM buffer of choice (mM^−1^cm^−1^)] and d (pathlength in 96 well plate: 0.5785 cm):


$$\begin{array}{r} Volumetric\;activity\;\left[UmL^{-1}\right]=\frac{\text{Δ}Absorbance\;.\;Vol_{End}\;.\;df}{\varepsilon \;.\;d\;.\;Vol_{Enzyme}}\\ Specific\;activity\;\left[Umg^{-1}\right]=\frac{Volumetric\;activity}{C_{Enzyme}} \end{array}$$

Based on ABTS activity assay, different ABTS stock concentration of 0.5, 1, 5, 7.5, 10, 12.5, 15, and 20 mM were used and measured for 4 min. The calculated specific activity values from triplicates of each concentration at 37°C and the respective pH were plotted against its corresponding substrate concentration yielding the Michaelis-Menten saturation curve. Kinetic values were determined via non-linear least square fitting.

### 2.5 AFB1 degradation assays and enzyme kinetics

AFB1 concentration used for the determination of kinetic values of BsCotA_Strep ranged from 50 to 15,000 μgL^−1^, with the higher levels set around the estimated solubility limits in water (PubChem). The reactions containing different BsCotA_Strep concentration (0.1, 0.02, 0.01 μM) were conducted in triplicates at 37°C in 100 mM sodium phosphate pH 7 with acetonitrile to a final concentration of 2%. After starting the reaction via enzyme addition, samples were drawn at 0, 1, 2, 3, 4, 5, 6, and 24 h. The reaction was stopped by addition of 99% ACN, diluting the sample to an ACN end concentration of 30%. Samples were further diluted with 30% ACN resulting in total sample dilution of 1:3. After centrifugation (14,000 rpm, 10 min), samples were stored at −20°C for following HPLC-FLD analysis. Kinetic parameters were generated from data obtained within the first 4 h for each data series and plotted against the measured starting concentration. These data were used to determine kinetic values using non-linear least square fitting and weighted least square regression models obtained in R (R Core Team, 2014) based on which specific activities were calculated.

Assays (total volume 1,280 μl) for the determination of the reaction products were conducted similarly at 37°C using 0.2 μM of BsCotA_Strep, and 5 μg/mL of AFB1 (experiment in duplicate) and 1 μg/mL AFQ1 in sodium phosphate buffer (100 mM, pH 7) as time course experiments with extended sampling (180 μl) intervals (4, 24, 48, and 72 h). The acetonitrile concentration in the reaction was fixed to 1%. In parallel, controls including 5 ppm of AFB1 and 1 ppm of AFQ1 as well as the BsCotA_Strep itself without the presence of toxin were incubated under identical conditions. After enzyme inactivation resulting in 1:1.4 dilution to 30% ACN, samples were centrifugated (14 k rpm, 10 min) and stored at −20°C for HPLC-FLD and HPLC-MS analysis as described below. In a further experiment, 5 ppm of AFQ1 and epi-AFQ1 were treated with and without BsCotA_Strep and samples were collected as described above. After dilution to 30% ACN and centrifugation (14,000 rpm, 10 min) samples were stored at −20°C for HPLC-MS and HPLC-QTOF analysis.

The pH profile of BsCotA_Strep on AFB1 was determined at pH 3, 7, and 8. The assays were conducted in duplicates at 37°C using 0.01 μM BsCotA_Strep and 9 μg/mL AFB1. Acetonitrile concentration was fixed to 1%. Samples were drawn at 0, 2, 4, 24, and 48 h. After enzyme, samples were centrifuged (14,000 rpm, 10 min) stored at −20°C for HPLC-FLD analysis.

### 2.6 HPLC-FLD analysis

AFB1 levels were routinely checked using a 1290 Infinity II HPLC system coupled to a FLD detector (Agilent, Waldbronn, Germany). Six microliter of the samples were injected and separated at 40°C on a Kinetex Biphenyl, 1.7 μm, 30 × 2.1 mm column (Phenomenex, Torrance, CA, USA; Part-Nr: 00A-4628-AN) coupled to a Kinetex UHPLC biphenyl 2.1 mm precolumn (Phenomenex, Torrance, CA, USA; Part-Nr: AJ0-9209). The 3.5 min method (flow rate 1 mL/min) comprised a gradient of eluent A with 5% methanol, 95% water and 0.1% acetic acid and eluent B with 100% methanol and 0.1% acetic acid, starting from a 70% A:30% B ratio and ending with 25% A:75% B, followed by 40 seconds of column equilibration with the initial ratio at the end of the run. Excitation/emission wavelengths for detecting the analyte were 365 and 460 nm, respectively. Data was evaluated against AFB1 standard series in similar concentrations of solvent using the OpenLab CDS 2.4 software (Agilent, Waldbronn, Germany).

### 2.7 HPLC-MS analysis

Detection and semi-quantification of AFB1 and putative reaction products were performed on a QTrap 5500 MS/MS system (Applied Biosystems, Foster City, CA, USA) equipped with a TurboV electrospray ionization (ESI) source and a 1290 Infinity I HPLC series (Agilent, Waldbronn, Germany). Samples were thawed at room temperature and vortexed. Three microliter were injected and separated at 25°C on a X-Bridge C18-column (150 × 2.1 mm i.d., 3.5 μm particle size, Waters, Milford, MA, USA) coupled to a pre-column (C18, 4 × 3 mm i.d., Security Guard Cartridge, Phenomenex, Torrance, CA, USA; Part-Nr: AJO-4287). Eluents comprised water with 0.1% formic acid (FA, v/v) as eluent A and methanol (0.1% FA, v/v) as eluent B at a 250 μL/min flow rate. The 45 min gradient used, started at 10% eluent B for 2 min, followed by linear increase to 100% within 30 min and hold for 5 min at 100% eluent B going back to 10% eluent B in 8 min. The LC-stream was directed into the mass spectrometer between 2.0 and 33 min. Q1 scan data (scan rate 1,000 Da/s) were recorded in the positive and negative ionization mode in the mass range between 100 and 850 Da. ESI source (Turbo Spray) depending on the ionization mode were: source temperature 500°C, curtain gas (CUR) 40 psi, nebulizer gas (GS1) and heater gas (GS2) 50 psi, ion-spray voltage ±4,500 V, declustering potential (DP) ±110 V, entrance potential (EP) ± 10. Data capture and analysis was performed using Analyst 1.6.2 (Sciex, Foster City, CA, USA). A quantitation method (details not shown) extracting a defined mass range from the full scan data collected in positive and negative Q1 scan mode was used for semi-quantification of the samples and data evaluation was based on quadratic, 1/ × weighted, calibration curves. Solutions for calibration were in the range of 25–3,000 ppb for AFB1 and 25–1,000 ppb for AFQ1 in 30% acetonitrile. Assuming similar ionization behavior, epi-AFQ1 was quantified based on the AFQ1 calibration data. Signal matrix enhancement or suppression was not evaluated.

### 2.8 HPLC-QTOF measurements

For QTOF-MS/MS calibration purposes, reserpine (m/z 609, 28,066) included in the ESI Positive Calibration Solution for the SCIEX X500R System (SCIEX Framingham, MA) was used. Analysis was carried out on a 1290 Infinity II HPLC series coupled to a QTOF-MS system X500R, SCIEX (Framingham, MA) equipped with an electrospray ionization (ESI) turboV^TM^ source operated in the positive and negative ESI mode. TOF-MS over an m/z range from 100 to 1,000 and TOF-MS/MS data were acquired. The sprayer probe includes an independent channel for the delivery of a calibration solution (reserpine), that allows to correct any drift in the mass accuracy of the mass analyzer. This calibration was run every five samples during the batch analysis. Source conditions were: ion spray voltage: 5,500 V; source temperature: 500°C; nitrogen gas flows (GS1 and GS2): 50 psi; and curtain gas: 35 psi, Ionspray voltage (V): 5,500, CAD gas: 7, Accumulation time (s): 0.15, Declustering potential (V): 80, Collision energy (V): 10. Chromatographic conditions (column, eluents, gradient etc.) remained as already described for the LC-MS analysis. However, here only 1 μl of each sample was injected. For qualitative and quantitative data processing Sciex O.S. software V 1.5 (SCIEX) was used.

### 2.9 Mutagenicity tests

The genotoxic potential of AFB1 and AFQ1 was assessed using the SOS Chromotest (EPBI, Ontario, Canada). All required materials including lyophilized cells and S9 homogenate were provided by the manufacturer. The assay was conducted following the provided instructions. Briefly, serial dilution of all analytes was assessed in triplicates in a range from 100 μg (10 ppm) to 625 ng. Ten microliter of the analyte in 50% acetonitrile and 100 μL of the bacterial suspension and S9 mixture were incubated for 2 h at 37°C in 96-well microtiter plates (Greiner Bio-one, Austria). Fifty microliter were transferred to a new plate containing 50 μL of the substrates. The plates were incubated at 37°C and measurements were taken at 410 nm (X-Gal)/600 nm (pNPP) in regular intervals on a Synergy H1 plate reader (BioTek, VT, USA).

## 3 Results and discussion

### 3.1 Optimization of the BsCotA expression strategy

Laccase CotA from *B. subtilis* (BsCotA) is a structural component of the endospore coat of the Gram-positive bacterium and has only 65% identity and 78% homology to CotA from *Bacillus licheniformis*. In contrast to CotA from *B. licheniformis*, considerably less is known about recombinant production for decomposition of the mycotoxin AFB1 by BsCotA and about underlying mechanisms. Therefore, in this study, the expression of BsCotA was optimized before investigation of decomposition of AFB1, which is in contrast to a substantially different CotA laccase from *B. licheniformis*, which was applied as wild-type enzyme in the reaction. Laccases are copper-dependent enzymes and therefore, it is extremely important that the catalytic metal atom is properly incorporated into the enzyme. When choosing the expression conditions, care must therefore be taken to ensure that Cu^2+^ is sufficiently available in *E. coli*. However, also other expression parameters are important for production of a fully functional enzyme. During overexpression, the folding and solubility of the enzyme is significantly influenced by the translation rate, which in turn might depend on the codon usage of the coding gene in the expression host. In addition, short peptide tags can increase the solubility of proteins and provide additional purification benefits.

For investigation of the expression parameters, BsCotA was produced based on the naturally occurring gene on the one hand and, on the other hand, using a codon usage-adapted gene for expression in *E. coli*. Subsequently, a codon-optimized gene for the expression of BsCotA with a C-terminal StrepTag (BsCotA_Strep) was designed, which not only enabled rapid purification by affinity chromatography, but might also contribute to better solubility of the enzyme. Expression of BsCotA_Strep was performed with and without addition of 0.25 mM CuCl_2_. To further improve the Cu^2+^ availability in the *E. coli* cells, the oxygen supply during the enzyme induction was reduced since it is known that intracellular levels of the cytotoxic Cu^2+^ are maintained in *E. coli* at low levels by an elaborate efflux system, resulting in low holoprotein titers (Rensing and Grass, 2003). To increase the ratio of functional protein, the induced culture was maintained at reduced oxygen supply, which led to highly improved cytoplasmic copper levels (Durão et al., 2008). The influence of the expression conditions on the enzyme quality was assessed based on the kinetic parameters of the purified enzymes with ABTS at pH 7 and 37°C as these conditions would meet the application conditions in the food and feed industry (Table 1).

**Table 1:** Kinetic parameters of BsCotA laccase variants on ABTS at pH 7 and 37°C.

**Enzyme**	**Codon usage optimization**	**Aeration**	**Addition CuCl_2_**	***V*_max_ [U/mg]**	***K*_m_ [mM]**	***k*_cat_ [s^−1^]**
BsCotA	No	Microaerobic	Yes	8.84 ± 0.18	0.22 ± 0.01	9.58 ± 0.20
BsCotA	Yes	Microaerobic	Yes	15.21 ± 0.22	0.19 ± 0.01	16.48 ± 0.24
BsCotA_Strep	Yes	Microaerobic	Yes	33.56 ± 2.02	0.78 ± 0.03	36.36 ± 2.19
BsCotA_Strep	Yes	Microaerobic	No	1.38 ± 0.12	0.50 ± 0.10	1.49 ± 0.13
BsCotA_Strep	Yes	Aerobic	Yes	0.30 ± 0.02	0.53 ± 0.09	0.33 ± 0.02

All experiments were carried out in triplicates.

**Figure 1:**
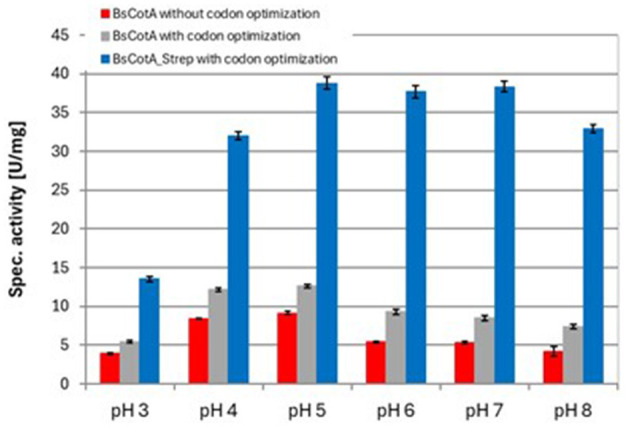
Determination of pH optima for different variants of the BsCotA laccase with ABTS at 37°C. Experiments were performed in duplicates using 0.01 μM BsCotA_Strep. BsCotA: laccase CotA from *Bacillus subtilis*.

As seen in Table 1, the codon usage optimization for *E. coli* led to an almost doubling of *V*_max_ and *k*_cat_ in the case of the untagged BsCotA whereas *K*_m_ remained almost unchanged. The higher activity could be due to better folding as a consequence of improved translation through optimized codons. In the case of BsCotA_Strep, the influence of the tested parameters became even more visible. Compared to BsCotA_Strep from a fully aerated expression culture, the micro-aerobe culture with addition of CuCl_2_ showed about 100x increased activity against the standard substrate ABTS and demonstrated the distinctive blue color indicating the inclusion of the T1 copper atom. Addition of CuCl_2_ during expression further improved the kinetics due to higher incorporation of the metal atom into the laccase. The intramolecular copper levels were photometrically determined from a complex formed with BCA. The readings at both wavelengths, 359 and 562 nm, established a ratio of statistically 1.8 Cu atoms per BsCotA_Strep molecule. Adding additional CuCl_2_ after enzyme purification did not significantly improve this ratio. The higher activity could be due to the gentler conditions during purification by affinity chromatography or to increased solubility due to the Strep tag. Remarkably, BsCotA_Strep yielded 26% more biomass in comparison to the expressed wild-type enzyme, which allowed production of 6.6 mg BsCotA_Strep/g biomass. Interestingly, fusion of the C-terminal StrepTag led to a shift in pH optima from 4 to 5 determined for the untagged BsCotA to a broadened optimal range of pH 5–7 (Figure 1). In comparison, Guo et al. (2020) determined the kinetic constants *K*_m_ = 34.9 μM and *k*_cat_ = 9.4 s^−1^ for CotA from *B. licheniformis* using ABTS at optimal conditions which were pH 4.2 and 80°C. However, the authors fused the laccase with a HisTag that could have leached out the copper from the enzyme. Consequently, they had to use significantly more enzyme in the reactions with AFB1.

For degradation of AFB1, longer reaction times were expected. However, the activity of BsCotA_Strep against ABTS diminished to 25% of the initial velocity within 24 h. In contrast, almost all the activity was retained in similar assays including varying amounts of BSA added to the reaction buffer or storage buffer. To limit any effects of BSA on AFB1 due to competitive binding, BSA was only added to the BsCotA_Strep solution in concentrations of 2 mg/mL, yielding an effective BSA concentration in assay of 0.7 μg^*^mL^−1^ (0.01 μM).

### 3.2 Degradation of AFB1 and characterization of reaction products

BsCotA_Strep was investigated for the ability to metabolize AFB1 without the presence of a mediator which would further add cost and complexity for industrial applications. The reaction kinetics was determined in three experiments with enzyme concentrations of 0.01, 0.02, and 0.1 μM using increasing concentrations of AFB1 up to the limit of reported solubility in water. Up to the highest concentration of 15,000 μg L^−1^ AFB1, the reaction velocity calculated from the first 4 h of incubation increased linearly, indicating that substrate saturation for AFB1 to reach *V*_max_ is above solubility in water. Instead, specific activities have been determined from data sets obtained with 0.1 and 0.02 μM BsCotA_Strep. Based on a weighted linear fit of the reaction velocity at different substrate levels, velocity did not scale relatively with increased enzyme concentrations. The specific activity at 500 μg/L—a representative high contamination level in feed—with 0.2 μM BsCotA_Strep was determined at 10.75 μg/Lh (0.17 nmol^*^min^−1*^mg^−1^) and 20.11 μg/Lh (0.35 nmol^*^min^−1*^mg^−1^) with 0.1 μM BsCotA_Strep. Comparison with data from literature is very difficult since unequal concentrations of AFB1 and laccase have been used for the reactions and applied at various pH and temperature conditions. Sun et al. (2023), for instance, incubated 40 μg of *B. licheniformis* CotA in 0.5 ml reaction volume at pH 7.5 and 37°C with 2 μg/ml AFB1 and detected 90% AFB1 decrease after 24 h of incubation which corresponds to a much lower turnover rate compared to BsCotA_Strep.

**Figure 2:**
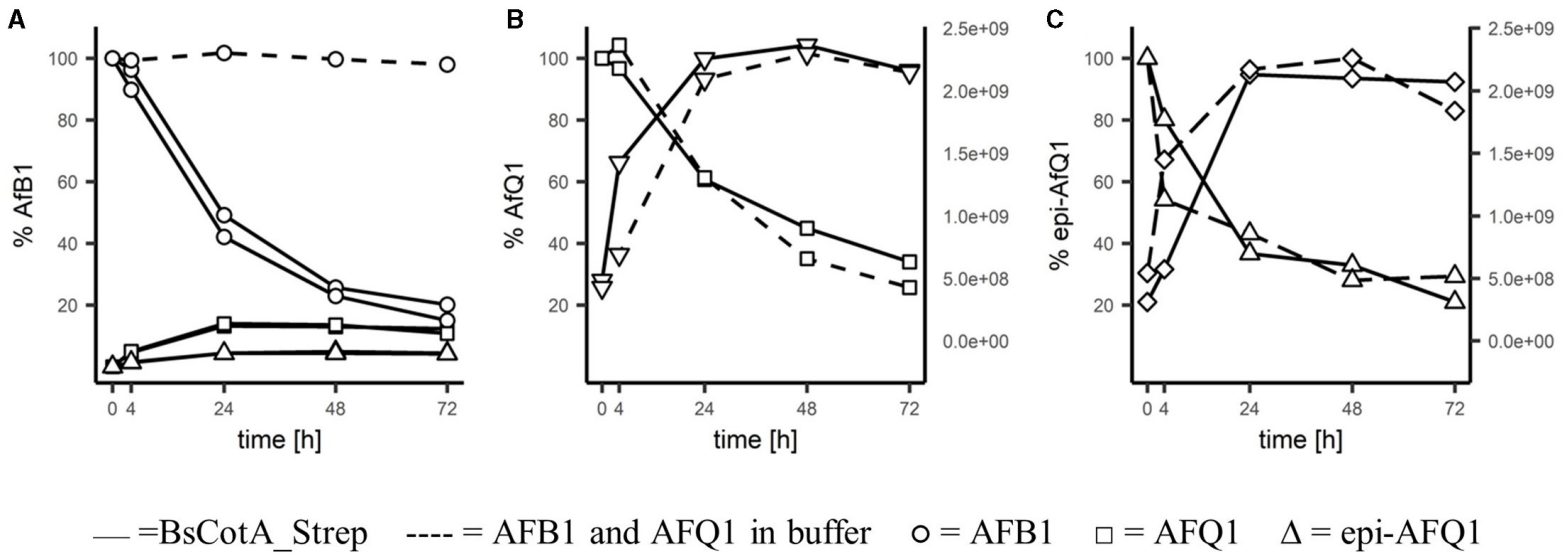
Degradation of AFB1 by the BsCotA_Strep laccase monitored using HPLC/MS. 5 ppm of **(A)** AFB1(circle symbol), **(B)** AFQ1 (square), **(C)** epi-AFQ1 (triangle) were each incubated with enzyme (full line) or without (dashed). Increase of instability products of AFQ1 (inverted triangle) and epi-AFQ1 (diamond) are shown as increases in peak areas (second *y*-axis).

For identification of the reaction products, 0.2 μM BsCotA_Strep was incubated with 5,000 μgL^−1^ of AFB1 at T = 37°C in sodium phosphate buffer (100 mM, pH 7). LC-MS analysis and comparison with the commercially available standards revealed a decrease of AFB1 and appearance of AFQ1 and epi-AFQ1 (Figure 2). The retention time was determined to be 17.5 min, 14.90 and 14.35 min for AFB1, AFQ1, and epi-AFQ1, respectively. Prolonged incubation led to losses in AFB1 recovery of up to 80% within 48 h at a starting concentration of 5,000 μgL^−1^ AFB1 and 0.2 μM BsCotA_Strep (Figure 2A). The AFB1 standard in phosphate buffer (100 mM, pH 7) without BsCotA_Strep did not reveal any significant changes in this time frame. The recovered concentrations were below the nominal AFB1 concentration (4,200 μgL^−1^ instead of 5,000 μgL^−1^ at the starting time point), which may be owed to matrix effects that were not further evaluated.

HPLC-FLD analysis showed similar concentration ranges and relative increases/decreases in aflatoxin abundances (data not shown) and the relative changes in time of the analyte remained accurate regardless of the total recovered concentrations. AFQ1 was the predominant product with 365 μgL^−1^ formed after 72 h compared to 131 μgL^−1^ epi-AFQ1 at this timepoint. The recovery of the initial AFB1 concentration in form of the detected metabolites was low, but this could be at least partly explained e.g., by the instability and rapidly degradation of the detected metabolite itself.

AFQ1 standard in acetonitrile/water (20/80) solution was shown to be stable. However, AFQ1 and epi-AFQ1 incubated in phosphate buffer were shown to be unstable and only 29 and 26% of 5,000 μgL^−1^ of either remain after 72 h of incubation. With the decline of substance, the accumulation of single products of instability for AFQ1 and epi-AFQ1 at RT 10.24 and 9.9 min, respectively, were observed (Figures 2B, C). Addition of BsCotA_Strep to AFQ1 or epi-AFQ1 did not lead to markedly different degradation slopes or additional reaction products in the full scan mass spec data, thus we do not consider AFQ1 and epi-AFQ1 substrates of BsCotA_Strep.

**Figure 3:**
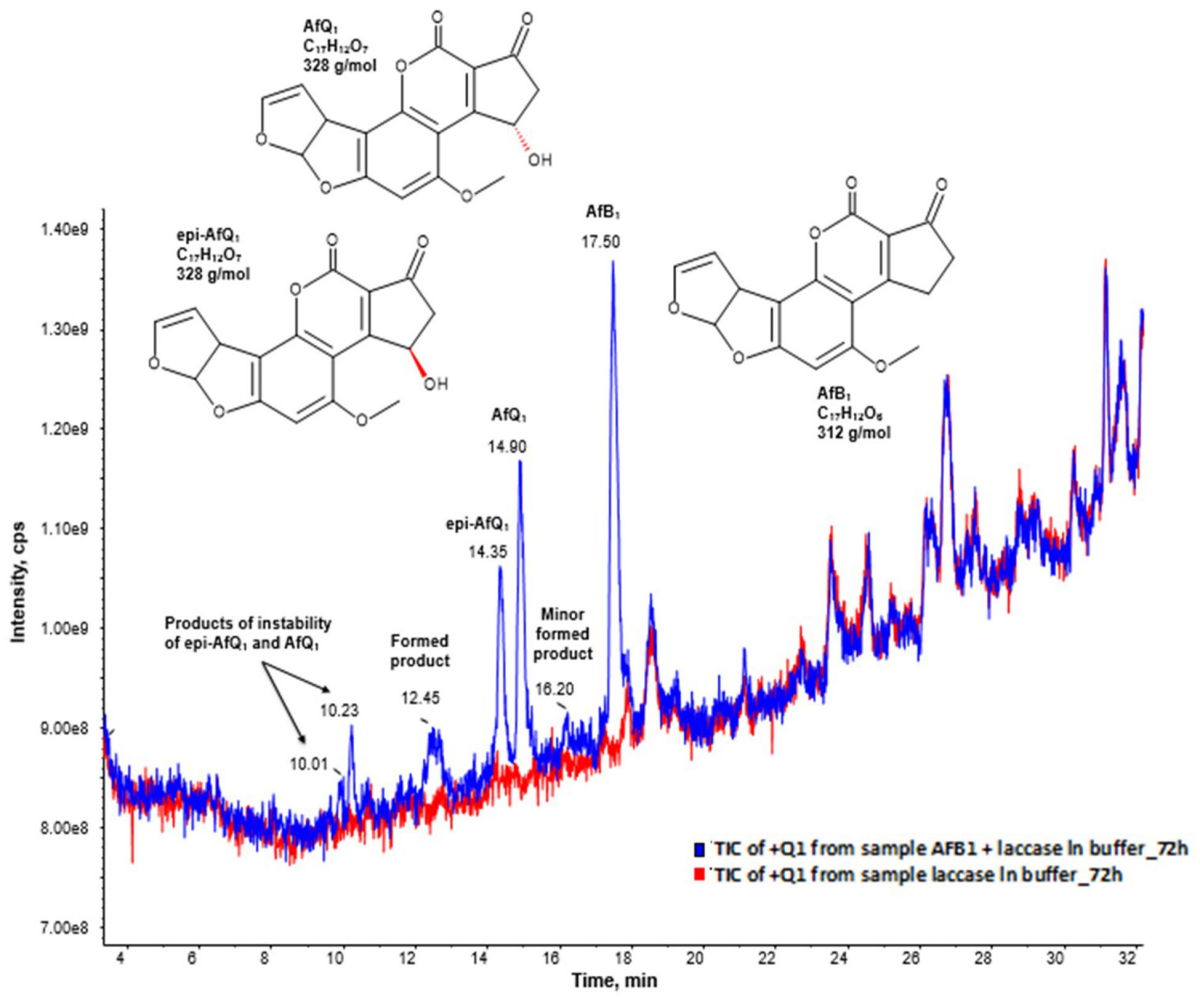
AFB1 degradation products after enzymatic treatment. Total ion chromatogram (TIC) of the Q1 scan in positive ionization mode after 48 (blue) hours of incubation with BsCotA_Strep. The blue curve displays the sample of AFB1 after enzymatic treatment and the red curve the sample of the laccase in buffer without AFB1 after 72 of incubation. The peaks 14.35, 14.90, and 17.50 have their origin to epi-AFQ1, AFQ1, and AFB1. The peaks at 10.23 min and 10.01 min have their origin to the instability of AFQ1 in the buffer used for incubation.

Moreover, the full scan mass spectra of the AFB1 samples incubated with BsCotA_Strep revealed the formation of putative reaction products at RT 10.01, 10.23, 12.45, 14.35, 14.90, and 16.20 min (Figure 3), with eventually potential minor reaction products remaining probably undetected. AFB1 appeared at a retention time of 17.50 min. The predominant formed products eluted at retention times 14.90 and 14.35 min and were by the comparison to the respective standards unambiguously assigned to AFQ1 and epi-AFQ1, respectively. The peak at 10.23 min was the respective product of instability AFQ1 in the buffer medium used for incubation, whereas the peak at 10.01 is the respective product of instability of epi-AFQ1.

Based on the collected LC-MS data—after background subtraction in the resulting full-scan mass spectrum of AFB1 in the pos. ionization mode—we could observe the formation of ions at m/z 313, 335, 642, and 647 which were assigned to [M+H]+, [M+Na]+, [2M+NH4]+, and [2M+Na]+, respectively, while in the neg. ionization mode mainly formation of [M-H]- at m/z 311 could be observed. After background subtraction the full-scan mass spectrum in the pos. ionization mode of AFQ1 and epi-AFQ1 revealed identical ions with the following m/z: [M+H]+ = 329, [M+NH4]+ = 346, [M+Na]+ = 351, [M+K]+ = 367, [2M+ NH4]+ = 674, [2M+Na]+ = 679. In the Q1 spectrum in the neg. ionization mode we observed the ions [M-14]- = 313, [M-H]- = 327, [M+HCOOH-H]-= 373.

For both peaks at 10.23 min and 10.01 min the assignment of the most intense ions was not univocal. In order to clarify this observation and to also determine the sum formula of these products we conducted additional HR-MS measurements. We assigned as [M-H-CO2]- = 301 and [M-H]- = 345 (negative ionization mode) and [M+H]+ = 329, [M+Na]+ = 351, [M+K]+ = 367, [2M+Na]+ = 679 (positive ionization mode). However, then the observed ions with m/z 347 and m/z 369 could not be assigned. Another assignment of the signals could be that [M+H]+ = 347 and [M+Na]+ = 369. Then by combination with the mass spectrum data obtained in the neg. ionization mode the peak at 10.23 min would have a MW of 346 g/mol.

The peak at 12.45 could be assigned to BsCotA_Strep itself while the origin of the peak at 17.50 remains elusive. It was assigned to M = 316 (observed mass signals in pos. ionization mode were at 317 [M+H+]+ and in neg. ionization mode at m/z 271.3 [M-CO2-H-]- and 315.3 [M-H-]-).

Molecular weights of +18 suggest the addition of water. Hydration of the vinyl ether in the terminal furan ring under strong acidic conditions are described for AFB1, AFG1, and AFM1 forming the AFB2a, AFG2a, and AFM2a (Martínez-Ruiz et al., 2018; Fuentes D et al., 2018). Such a reaction forming the undescribed (epi)-AFQ2a should be possible for (epi-)AFQ1 as well. To test whether AFQ2a is identical with the instability product of AFQ2, we diluted 20 μL of a 125 μgL^−1^ AFQ1 solution to 250 μL with 2.5 N HCl and analyzed the solution after overnight incubation. Ion source fragments of AFQ2a captured at RT 10.01, did however not match fragmentation patterns observed for the instability product of AFQ1 with this retention time RT.

AFQ1 and epi-AFQ1 were also reported by Guo et al. (2020) as main degradation products of AFB1 after incubation with CotA, determined by LC-MS, NMR, and CD. However, they applied CotA from *B. licheniformis* ANSB821 in their study. Mutagenesis of the enzyme led to the identification of mutant Q441A, which had a 1.73-fold higher catalytic efficiency (*k*_cat_/*K*_m_) than the wt enzyme (Liu et al., 2023). Similarly, CotA from *B. licheniformis* ZOM-1 was heterologously expressed in *Escherichia coli* (*E. coli*) and investigated for its activity on AFB1 (Sun et al., 2023). Here, the degradation products AFB1- diol, AFQ1 or epi-AFQ1 were identified by UPLC-HRMS/MS. However, none of these studies mentioned and discussed the instability of the degradation products.

### 3.3 Genotoxicity assessment of AFQ1

The genotoxicity assay relies on the quantitative de-repression of the bacterial SOS response to DNA damage. The test employs an engineered *E. coli* strain, which expressed lacZ—the structural gene for ß-galactosidase—in fusion with the SOS-responsive sfiA, involved in regulating cell division. Thus, the genotoxicity of target compounds can be directly determined in a photometric assay measuring LacZ activity (substrate X-Gal). Endogenous phosphatase released from damaged cells is determined simultaneously with *para*-nitrophenylphosphat (pNPP) as substrate to assess additional cell toxicity. Compounds such as aflatoxins require metabolic activation by hepatic cytochrome P450 oxidoreductases. To this end S9 rat-liver homogenate including NADPH and a corresponding enzymatic regeneration system are added to the assay.

AFQ_1_ is expected to be less or even non-toxic according to Hsieh et al. (1974), who have used the Ames test with a single indicator strain (*Salmonella typhimurium* TA1538). To our knowledge no experiments have been conducted to clearly explain the reduced toxicity of AFQ1. The laccase facilitates the oxidation of the C-14 position. This additional hydroxyl mojety could reduce toxicity by either limiting the substrate affinity of liver monooxygenases that activate AFB1 by epoxidizing the 8,9-furan ring, and/or its increased hydrophilic properties and steric alterations reduce successful migration between DNA sheets.

To reassess AFQ_1_ toxicity with the SOS Chromotest, we included a range of dilutions in acetonitrile up to 10 ppm and compared readings to controls including only the solvent at both wavelengths, yielding a “SOSIF” value corrected for background activity:


$$\begin{array}{l} SOSIF=\frac{\left(OD600,\;i\right)/\left(OD420,i\right)}{\left(OD600,negative\right)/\left(OD420,negative\right)} \end{array}$$

**Figure 4:**
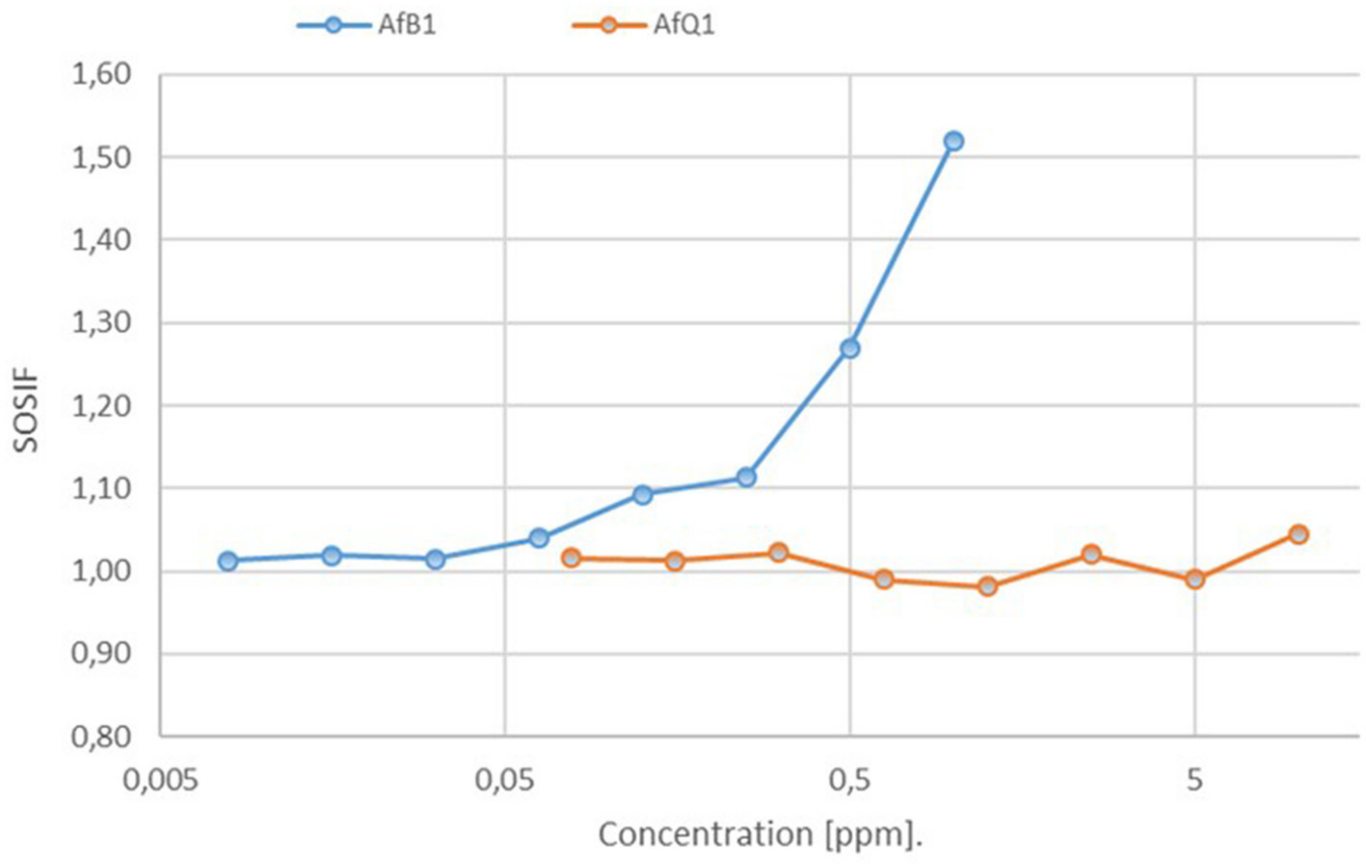
Assessment of the genotoxic potential of AFB1 and AFQ1 by the SOS Chromotest based on the measurement of the primary response of *E.coli* cells to genetic damage. Estimated SOSIF values for AFB1 against AfQ1 resulting after decomposition by laccase. SOSIF, SOS induction factor.

Similar measurement on dilutions were conducted with the positive control, 4-nitroquinoline 1-oxide (4-NQO, up to 10 ppm) and AFB_1_ (up to 1 ppm) in three technical replicates. The recorded SOSIF values for the positive control 4-NQO (not shown) and AFB1 show a clear dose-depending response (Figure 4). In comparison for AFQ1 no significant changes were observed between the individual concentrations up to 10 ppm. The observed values compare to a AFB1 concentration of 0.125 ppm. We therefore conclude that 10 ppm AFQ1 is at best at the toxicity level of 0.125 ppm AFB1. Its genotoxity is at 80-times lower (10/0.125) when compared to the toxicity of AFB1 according to the measurements.

The results are in accordance with Hsieh et al. (1974) and have been evaluated in the past also by other authors using various methods. Wang et al. (2019b) used growth of hydra in a culture containing AFB1 treated or non-treated with one of the BsCotA/mediators and observed that the hydra collapsed after 18 h when incubated with non-treated AFB1 whereas the hydra remained alive when they were incubated with AFB1 treated with BsCotA in the presence of methyl syringate. Guo et al. (2020) treated human liver cells (L-02) with AFQ1 and epi-AFQ1 and observed that the viability of the cells decreased with increasing concentrations of AFB1 from 0 to 100 μM after incubation for 48 h whereas the cells were not reduced after exposure to AFQ1 or epi-AFQ1. According to further studies, AFQ1 is approximately eighteen times less toxic and approximately eighty-three times less mutagenic than AFB1 (Popescu et al., 2022).

## 4 Conclusion

The contamination of feed and food with mycotoxins is a major health problem for humans and animals, which is predicted to worsen due to climate change (Leggieri et al., 2021). In addition to the high health risk, contamination with mycotoxins also causes considerable economic losses for the food and animal feed industry. The detoxification of mycotoxins has therefore become a major problem that could be reduced by biological methods such as enzymes, as they are highly specific and can work under environmentally friendly conditions. Oxidative enzymes such as laccases are designed from nature to transform highly complex molecules due to their high oxidative potential and have also shown activity on aflatoxin B1. Laccases use molecular oxygen as a co-substrate and produce only water as a by-product, which makes their application environmentally friendly. In addition, they can be produced in large quantities and at low cost. Laccases are therefore already used in various processes in the food industry, for example, to stabilize wine and beer, or to give doughs in the baking industry the necessary strength and stability. In this study, we examined the activity of the laccase CotA from *Bacillus subtilis* on aflatoxin B1 and elucidated for the first time the reaction products of this laccase, which has only 65% identity to CotA from *B. licheniformis*, using MS analysis. For this purpose, BsCotA was optimized with regard to the maximum incorporation of the catalytically active copper atoms leading to substantially more active enzyme. The optimized BsCotA was incubated with AFB1 and the reaction products AFQ1 and epi-AFQ1 were identified by MS. The fact that the conversion to the less toxic metabolites takes place without the addition of mediators makes the use of laccase in food and feed particularly attractive. The recovery of the initial AFB1 concentration in the form of detected metabolites was investigated in more detail as well as instability of the metabolites was demonstrated, which were no substrates for BsCotA. Unambiguous identification of the reaction products is an important topic as themselves may be equally or even more toxic than the parent toxin. Therefore, both metabolites were examined in a genotoxicity test and an 80-fold lower toxicity was found compared to AFB1. Due to the conversion of AFB1, the laccase could have the potential to convert other toxic secondary metabolites and thus represent a broader approach for the biological detoxification of mycotoxins in the future. In further studies, however, the specificity and turnover rates of the laccase should be improved in order to successfully degrade the strongly matrix-associated aflatoxins at very low concentrations.

## Data Availability

The datasets presented in this study can be found in online repositories. The names of the repository/repositories and accession number(s) can be found in the article/supplementary material.

## References

[B1] Aguilar F. Hussain S. P. Cerutti P. (1993). Aflatoxin B1 induces the transversion of G → T in codon 249 of the P53 tumor suppressor gene in human hepatocytes. Proc. Natl. Acad. Sci. U. S. A. 90, 8586–8590. 10.1073/pnas.90.18.8586 8397412 PMC47402

[B2] Alberts J. F. Gelderblom W. C. A. Botha A. van Zyl W. H. (2009). Degradation of aflatoxin B1 by fungal laccase enzymes. Int. J. Food Microbiol. 135, 47–52. 10.1016/j.ijfoodmicro.2009.07.022 19683355

[B3] Al-Rajhi A. M. H. Ganash M. Alshammari A. N. Alsalamah S. A. Abdelghany T. M. (2024). *In vitro* and molecular docking evaluation of target proteins of lipase and protease for the degradation of aflatoxins. BioResources 19, 2701–2713. 10.15376/biores.19.2.2701-2713

[B4] Arimboor R. (2024). Metabolites and degradation pathways of microbial detoxification of aflatoxins: a review. Mycotoxin Res. 40, 71–83. 10.1007/s12550-023-00515-0 38151634

[B5] Bashiri G. (2022). Cofactor F420, an emerging redox power in biosynthesis of secondary metabolites. Biochem. Soc. Trans. 50, 253–267. 10.1042/BST20211286 35191491

[B6] Brenner A. J. Harris E. D. (1995). A quantitative test for copper using bicinchoninic acid. Anal. Biochem. 226, 80–84. 10.1006/abio.1995.1194 7785783

[B7] Ciegler A. Lillehoj E. B. Peterson R. E. Hall H. H. (1966). Microbial detoxification of aflatoxin. Appl. Environ. Microbiol. 14, 934–939. 10.1128/am.14.6.934-939.1966 16349699 PMC1058446

[B8] Croy R. G. Essigmann J. M. Reinhold V. N. Wogan G. N. (1978). Identification of the principal aflatoxin B1-DNA adduct formed *in vivo* in rat liver. Proc. Natl. Acad. Sci. U. S. A. 75, 1745–1749. 10.1073/pnas.75.4.1745 273905 PMC392416

[B9] Durão P. Chen Z. Fernandes A. T. Hildebrandt P. Murgida D. H. Todorovic S.. (2008). Copper incorporation into recombinant CotA laccase from bacillus subtilis: characterization of fully copper loaded enzymes. J. Biol. Inorg. Chem. 13, 183–193. 10.1007/s00775-007-0312-0 17957391

[B10] Essigmann J. M. Croy R. G. Nadzan A. M. Busby W. F. Reinhold V. N. Büchi G.. (1977). Structural identification of the major DNA adduct formed by aflatoxin B1 *in vitro*. Proc. Natl. Acad. Sci. U. S. A. 74, 1870–1874. 10.1073/pnas.74.5.1870 266709 PMC431033

[B11] EUR-Lex - 02006R1881-20140701 - EN - EUR-Lex (2023). Commission Regulation (EU) 2023/915 on maximum levels for certain contaminants in food and repealing Regulation (EC) No 1881/2006. Off. J. Eur. Union L 119, 103–157.

[B12] Fouché T. Claassens S. Maboeta M. (2020). Aflatoxins in the soil ecosystem: an overview of its occurrence, fate, effects and future perspectives. Mycotoxin Res. 36, 303–309. 10.1007/s12550-020-00393-w 32270463

[B13] Fuentes D S. Carvajal M. Ruiz S. Martínez N. C. Azucena Gomez A. Rojo F. (2018). Presence of mutagens and carcinogens, called aflatoxins, and their hydroxylated metabolites in industrialized food for dogs. J. Microb. Biochem. Technol. 10:1000399. 10.4172/1948-5948.100039930468840

[B14] Giardina P. Faraco V. Pezzella C. Piscitelli A. Vanhulle S. Sannia G. (2010). Laccases: a never-ending story. Cell. Mol. Life Sci. 67, 369–385. 10.1007/s00018-009-0169-1 19844659 PMC11115910

[B15] Gu R. Yang X. Cheng L. Zhang Q. Li P. Mao J. (2024). Screening, identification, and application of bacillus velezensis NWPZ-8 probiotics to aflatoxin detoxification in peanut meal. ACS Food Sci. Technol. 4, 2155–2166. 10.1021/acsfoodscitech.4c00371

[B16] Guo Y. Qin X. Tang Y. Ma Q. Zhang J. Zhao L. (2020). Cota laccase, a novel aflatoxin oxidase from *Bacillus licheniformis*, transforms aflatoxin B1 to aflatoxin Q1 and epi-aflatoxin Q1. Food Chem. 325:126877. 10.1016/j.foodchem.2020.126877 32387986

[B17] Hsieh D. P. H. Salhab A. S. Wong J. J. Yang S. L. (1974). Toxicity of aflatoxin Q1 as evaluated with the chicken embryo and bacterial auxotrophs. Toxicol. Appl. Pharmacol. 30, 237–242. 10.1016/0041-008X(74)90095-7

[B18] Iyer R. S. Coles B. F. Raney K. D. Thier R. Peter Guengerich F. Harris T. M. (1994). DNA adduction by the potent carcinogen aflatoxin B1: mechanistic studies. J. Am. Chem. Soc. 116, 1603–1609. 10.1021/ja00084a001

[B19] Klvana M. Bren U. (2019). Aflatoxin B1–formamidopyrimidine DNA adducts: relationships between structures, free energies, and melting temperatures. Molecules 24:150. 10.3390/molecules24010150 30609733 PMC6337653

[B20] Kunz-Vekiru E. Subagia R. Wolfsberger D. Schatzmayr G. Ribitsch D. Guebitz G.. (2019). “Mycotoxins and phycotoxins conference in GRC,” in The B. Subtilis CotA Laccase Metabolizes Aflatoxin B1 into Aflatoxin Q1 and Epi-Aflatoxin Q1. (Stonehill College Massachusetts).

[B21] Leggieri M. C. Toscano P. Battilani P. (2021). Predicted aflatoxin B1 increase in Europe due to climate change: actions and reactions at global level. Toxins 13:292. 10.3390/toxins13040292 33924246 PMC8074758

[B22] Li J. Huang J. Jin Y. Wu C. Shen D. Zhang S.. (2018). Aflatoxin B1 degradation by salt tolerant tetragenococcus halophilus CGMCC 3792. Food Chem. Toxicol. 121, 430–436. 10.1016/j.fct.2018.08.063 30165130

[B23] Liu Y. Guo Y. Liu L. Tang Y. Wang Y. Ma Q.. (2023). Improvement of aflatoxin B1 degradation ability by *Bacillus licheniformis* CotA-laccase Q441A mutant. Heliyon 9:e22388. 10.1016/j.heliyon.2023.e22388 38058637 PMC10696099

[B24] Liu Y. Mao H. Hu C. Tron T. Lin J. Wang J.. (2020). Molecular docking studies and in vitro degradation of four aflatoxins (AFB1, AFB2, AFG1, and AFG2) by a recombinant laccase from *Saccharomyces cerevisiae*. J. Food Sci. 85, 1353–1360. 10.1111/1750-3841.15106 32220140

[B25] Loi M. Fanelli F. Zucca P. Liuzzi V. C. Quintieri L. Cimmarusti M. T.. (2016). Aflatoxin B1 and M1 degradation by Lac2 from pleurotus pulmonarius and redox mediators. Toxins 8:245. 10.3390/toxins8090245 27563923 PMC5037472

[B26] Loi M. Renaud J. B. Rosini E. Pollegioni L. Vignali E. Haidukowski M.. (2020). Enzymatic transformation of aflatoxin B1 by Rh_DypB peroxidase and characterization of the reaction products. Chemosphere 250:126296. 10.1016/j.chemosphere.2020.126296 32135437

[B27] Mariën K. Moyer R. Loveland P. Van Holde K. Bailey G. (1987). Comparative binding and sequence interaction specificities of aflatoxin B1, aflatoxicol, aflatoxin M1, and aflatoxicol M1 with purified DNA. J. Biol. Chem. 262, 7455–7462. 10.1016/S0021-9258(18)47588-6 2953721

[B28] Martínez-Ruiz N. C. Carvajal-Moreno M. Rojo-Callejas F. Fuentes-Daza S. Gómez-Carrión A. Ruiz-Velasco S. (2018). Mutagens and carcinogens called aflatoxins and their hydroxylated metabolites in food for domestic cats. Biochem. Anal. Biochem. 7:366. 10.4172/2161-1009.1000366

[B29] Martins L. O. Durão P. Brissos V. Lindley P. F. (2015). Laccases of prokaryotic origin: enzymes at the interface of protein science and protein technology. Cell. Mol. Life Sci. 72, 911–922. 10.1007/s00018-014-1822-x 25572294 PMC11113980

[B30] Mishra H. N. Das C. (2003). A review on biological control and metabolism of aflatoxin. Crit. Rev. Food Sci. Nutr. 43, 245–264. 10.1080/10408690390826518 12822672

[B31] Munk L. Andersen M. L. Meyer A. S. (2018). Influence of mediators on laccase catalyzed radical formation in lignin. Enzyme Microb. Technol. 116, 48–56. 10.1016/j.enzmictec.2018.05.009 29887016

[B32] Mwakinyali S. E. Ming Z. Xie H. Zhang Q. Li P. (2019). Investigation and characterization of myroides odoratimimus strain 3J2MO aflatoxin B 1 degradation. J. Agric. Food Chem. 67, 4595–4602. 10.1021/acs.jafc.8b06810 30907589

[B33] Neto B. A. D. Lapis A. A. M. (2009). Recent developments in the chemistry of deoxyribonucleic acid (DNA) intercalators: principles, design, synthesis, applications and trends. Molecules. 14, 1725–1746. 10.3390/molecules14051725 19471193 PMC6254398

[B34] Pardo I. Camarero S. (2015). Laccase engineering by rational and evolutionary design. Cell. Mol. Life Sci. 72, 897–910. 10.1007/s00018-014-1824-8 25586560 PMC4323517

[B35] Popescu R. G. Rădulescu A. L. Georgescu S. E. Dinischiotu A. (2022). Aflatoxins in feed: types, metabolism, health consequences in swine and mitigation strategies. Toxins 14:853. 10.3390/toxins14120853 36548750 PMC9783261

[B36] R Core Team (2014). R: A Language and Environment for Statistical Computing. Vienna: R Foundation for Statistical Computing. Available at: http://www.R-project.org/

[B37] Rensing C. Grass G. (2003). *Escherichia coli* mechanisms of copper homeostasis in a changing environment. FEMS Microbiol. Rev. 27, 197–213. 10.1016/S0168-6445(03)00049-4 12829268

[B38] Rushing B. R. Selim M. I. (2019). Aflatoxin B1: a review on metabolism, toxicity, occurrence in food, occupational exposure, and detoxification methods. Food Chem. Toxicol. 124, 81–100. 10.1016/j.fct.2018.11.047 30468841

[B39] Shimada T. Guengerich F. P. (1989). Evidence for cytochrome P-450(NF), the nifedipine oxidase, being the principal enzyme involved in the bioactivation of aflatoxins in human liver. Proc. Natl. Acad. Sci. U. S. A. 86, 462–465. 10.1073/pnas.86.2.462 2492107 PMC286490

[B40] Stone M. P. Gopalakrishnan S. Harris T. M. Graves D. E. (1988). Carcinogennucleic acid interactions:equilibrium binding studies of aflatoxins B1 and B2. J. Biomol. Struct. Dyn. 5, 1025–1041. 10.1080/07391102.1988.10506447 3152158

[B41] Sun F. Yu D. Zhou H. Lin H. Yan Z. Wu A. (2023). CotA laccase from *Bacillus licheniformis* ZOM-1 effectively degrades zearalenone, aflatoxin B1 and alternariol. Food Control 145:109472. 10.1016/j.foodcont.2022.109472

[B42] Tadesse M. A. D'Annibale A. Galli C. Gentili P. Sergi F. (2008). An assessment of the relative contributions of redox and steric issues to laccase specificity towards putative substrates. Org. Biomol. Chem. 6, 868–878. 10.1039/b716002j 18292878

[B43] Taheur F. B. Mansour C. Jeddou K. B. Machreki Y. Kouidhi B. Abdulhakim J. A.. (2020). Aflatoxin B1 degradation by microorganisms isolated from kombucha culture. Toxicon 179, 76–83. 10.1016/j.toxicon.2020.03.004 32345454

[B44] Tang Y. Liu X. Tang L. Dong J. (2024). Investigating the mechanism of *Bacillus amyloliquefaciens* YUAD7 degrading aflatoxin B1 in alfalfa silage using isotope tracing and nuclear magnetic resonance methods. Chem. Biol. Technol. Agric. 11:102. 10.1186/s40538-024-00619-4

[B45] Taylor M. C. Jackson C. J. Tattersall D. B. French N. Peat T. S. Newman J.. (2010). Identification and characterization of two families of F420H2-dependent reductases from mycobacteria that catalyse aflatoxin degradation. Mol. Microbiol. 78, 561–575. 10.1111/j.1365-2958.2010.07356.x 20807200 PMC3034190

[B46] Teniola O. D. Addo P. A. Brost I. M. Färber P. Jany K. D. Alberts J. F.. (2005). Degradation of aflatoxin B1 by cell-free extracts of rhodococcus erythropolis and *Mycobacterium fluoranthenivorans* Sp. Nov. DSM44556T. Int. J. Food Microbiol. 105, 111–117. 10.1016/j.ijfoodmicro.2005.05.004 16061299

[B47] Vanhoutte I. Audenaert K. De Gelder L. (2016). Biodegradation of mycotoxins: tales from known and unexplored worlds. Front. Microbiol. 7:561. 10.3389/fmicb.2016.00561 27199907 PMC4843849

[B48] Vekiru E. Fruhauf S. Rodrigues I. Ottner F. Krska R. Schatzmayr G.. (2015). *In vitro* binding assessment and *in vivo* efficacy of several adsorbents against aflatoxin B1. World Mycot. J. 8, 477–488. 10.3920/WMJ2014.180029510743

[B49] Wang J. Ogata M. Hirai H. Kawagishi H. (2011). Detoxification of aflatoxin B1 by manganese peroxidase from the white-rot fungus phanerochaete sordida YK-624. FEMS Microbiol. Lett. 314, 164–169. 10.1111/j.1574-6968.2010.02158.x 21118293

[B50] Wang X. Bai Y. Huang H. Tu T. Wang Y. Wang Y.. (2019a). Degradation of aflatoxin B1 and zearalenone by bacterial and fungal laccases in presence of structurally defined chemicals and complex natural mediators. Toxins 11:609. 10.3390/toxins11100609 31652557 PMC6832423

[B51] Wang X. Cui L. Liu M. Qi Z. Luo H. Huang H.. (2024). Theoretical insights into the mechanism underlying aflatoxin B1 transformation by the BsCotA-methyl syringate system. Ecotoxicol. Environ. Saf. 272:116049. 10.1016/j.ecoenv.2024.116049 38301584

[B52] Wang X. Qin X. Hao Z. Luo H. Yao B. Su X. (2019b). Degradation of four major mycotoxins by eight manganese peroxidases in presence of a dicarboxylic acid. Toxins 11:566. 10.3390/toxins11100566 31569657 PMC6833064

[B53] Wu J. Wang Z. An W. Gao B. Li C. Han B.. (2024). Bacillus subtilis simultaneously detoxified aflatoxin B1 and zearalenone. Appl. Sci. 14:1589. 10.3390/app1404158931652557

[B54] Xu F. Berka R. M. Wahleithner J. A. Nelson B. A. Shuster J. R. Brown S. H.. (1998). Site-directed mutations in fungal laccase: effect on redox potential, activity and PH profile. Biochem. J. 334, 63–70. 10.1042/bj3340063 9693103 PMC1219662

